# Robotic technology in surgery; a classification system of soft tissue surgical robotic devices

**DOI:** 10.1007/s00464-024-10861-4

**Published:** 2024-05-21

**Authors:** Brandon Cowan, Camilla Gomes, Paul Morris, Raymond Fryrear, William Petraiuolo, Matt Walton, Adnan Alseidi, Santiago Horgan, Monika Hagen

**Affiliations:** 1 Department of Surgery, University of California San Francisco, San Francisco, CA USA; 2 Johnson and Johnson MedTech, Santa Clara, CA USA; 3 Department of Surgery, University of California San Diego, San Diego, CA USA; 4 Department of Surgery, University Hospital Geneva, Geneva, Switzerland; 5 UCSF, East Bay Surgery, 1411 E 31stSt, QIC 22134, Oakland, CA 94602 USA

**Keywords:** Robotic surgery, Robotic-assisted surgery, Classification, Medical technology, Medical device

## Abstract

**Background:**

The field of robotic-assisted surgery is rapidly growing as many robotic surgical devices are in development and about to enter the market. Currently, there is no universally accepted language for labeling the different robotic systems. To facilitate this communication, we created what is, to our knowledge, the first classification of surgical robotic technologies that organizes and classifies surgical robots used for endoscopy, laparoscopy and thoracoscopy.

**Methods:**

We compiled a list of surgical robots intended to be used for endoscopy, laparoscopy, and/or thoracoscopy by searching United States, European, Hong Kong, Japan, and Korean databases for approved devices. Devices showcased at the 2023 Annual Meeting for the Society of Robotic Surgery were added. We also systematically reviewed the literature for any existing surgical robotic classifications or categorizations. We then created a multidisciplinary committee of 8 surgeons and 2 engineers to construct a proposed classification of the devices included in our search.

**Results:**

We identified 40 robotic surgery systems intended to be used for endoscopy, laparoscopy and/or thoracoscopy. The proposed classification organizes robotic devices with regard to architecture, port design, and configuration (modular carts, multi-arm integrated cart, table-attachable or arm-table integration).

**Conclusion:**

This 3-level classification of robotic surgical devices used for endoscopy, laparoscopy and/or thoracoscopy describes important characteristics of robotic devices systematically.

## Background

Many may equate a surgical robot, at least in the abdominal and thoracic space, with the da Vinci Surgical System (Intuitive Surgical, Inc, Sunnyvale, CA, US), a device which holds a virtual monopoly in robotic-assisted surgery in the Western markets [1]. The global economy for surgical robotics has grown to $7.9 billion USD in 2022 with projection for continued growth in the coming years, and nearly 900,000 robotic procedures across disciplines are being performed yearly in the USA, with many new players vying for market share [2, 3]. As an indicator of the growth in the industry, over 46 robotic platforms were showcased at the Society of Robotic Surgery Annual Meeting in July of 2023 [4]. The field of surgical robotics is experiencing an inflection point in growth with a steadily increasing number of robotic surgical systems entering clinical use or being close to market release. With a range of features, the robotic surgery market is likely to become more diverse, and consequently, more complex. This leaves the term “robotic-assisted surgery” open to interpretation regarding many different architectures and functions of a surgical robot.

The diversity of surgical robotics is creating an increasing unmet need for a common language for all users to assess, evaluate, and communicate effectively about surgical robotics. Complex subjects can be simplified by organizing knowledge into a classification system. The concept of a classification framework as an information science dates to the 18^th^ century, when it was introduced as a scientific attempt to classify organisms into a taxonomy [5]. Classification frameworks are commonplace in many disciplines with knowledge organization, including healthcare [6]. As such, it would be very helpful if surgical academic societies, researchers, engineers, and those in the medical technology industry shared a classification system to effectively communicate about the features, form, and function of surgical robots.

This project offers a classification of endoscopic, thoracoscopic, or laparoscopic surgery robotic systems based upon their architecture and defining features which is, to our knowledge, the first attempt to systematically classify robotic systems for soft tissue surgery.

## Methods

The National Information Standards Organization (NISO) has established guidelines for knowledge organization and construction of controlled vocabularies such as classification systems [7]. These guidelines were used to determine the methods of this project which includes the following steps:

1. Identification of existing endoscopy, laparoscopic, and thoracoscopy robotic systems
2. Literature search for existing surgical robotic technology and potential classification or taxonomy schemes
3. Creation of classification

### Identification of existing relevant robotic systems

First, we compiled a comprehensive list of surgical robots which are approved for sale by the governments listed hereafter. As of 9/11/2023, the FDA databases were searched systematically for filings of robotic-assisted surgical devices using FDA product codes; NEQ (device, telemedicine, robotic), NAY (system, surgical, computer-controlled instrument), EOQ (bronchoscope), FGB (ureteroscope), or HET (laparoscope, GYN, and accessories) [8, 9]. The European Commission European Database on Medical Devices was searched on 9/20/2023 with the search code “Z12020101: Robot-assisted endoscopic surgery systems” [10]. The Hong Kong Medical Device Administrative Control System, the Japanese Pharmaceuticals and Medical Devices Agency (JPMDA), and Korean Ministry of Food and Drug Safety (KMFDS) Medical Device Approval Report were each searched for the keyword “robot” or “robotic” [11–13]. Filings found in database searches were screened by title and submission text and if clarification of the function or form of the product was necessary, further information was gathered from the products’ webpages. Products were included if they had a robotic arm(s) for intended use in surgical procedures on humans and had made a filing between 2003 and the current day in 2023, except for KMFDS where data were not publicly available for devices approved prior to 2014. A further inclusion criterion was that the product must also be intended for use in endoscopy, laparoscopy, and/or thoracoscopy. Robotic surgical devices with intended use in orthopedic surgery, neurosurgery, and endovascular surgery without soft tissue or endoscopic applications were excluded.

To capture endoscopic, thoracoscopic, or laparoscopic surgery tissue robots which are publicly marketed but may not yet have had a Premarket Notification or Premarket Approval by the FDA, we investigated the program book of the July 2023 Annual Meeting for the Society of Robotic Surgery; if vendors with booths in the exhibition hall were advertising a surgical robot then the device was considered for study inclusion [14].

### Literature search for existing surgical robotic technology and potential classification or taxonomy schemes

PubMed was systematically reviewed in accordance with the Preferred Reporting Items for Systematic Rviews and Meta-analyses (PRISMA) 2020 statement [15]. PubMed was searched on 11/16/2023 with the terms [(“surgical robot*” OR “medical robot*” OR “robotic-assisted surgery” OR “robotic surgery”) AND (taxonomy OR classification)]. Search results were included if content included a review of surgical robotic technology and were excluded if content focused on a specific procedure, technique, or clinical outcome, if focused on an engineering feature, or if it did not address endoscopic, thoracoscopic, or laparoscopic surgery, i.e., were specific to neurosurgery, endovascular surgery, and orthopedic surgery. Additionally, articles were excluded if full text could not be identified or was not available in the English language. Included publications were analyzed for potential classification or taxonomy schemes to incorporate in our proposed comprehensive classification.

### Creation of classification

We applied a combination of committee and empirical approaches to create a classification with the devices that had been found in our systematic search [7]. We chose a combination approach as it best suited our panel of experts to draw relationships and then classify from the broadest category first, then to create increasing levels of specificity. Our multidisciplinary committee was comprised of 2 general surgery residents (CG, BC), 3 board-certified general surgeons (RF, MH and PM) and 2 robotic clinical engineers (BT, SL). Upon reaching a final consensus classification, further review was performed by 3 additional board-certified general surgeons (WP, AA, and SH). Drafts were first constructed by CG and BC, then reviewed by the group and iteratively adjusted until a consensus was met.

## Results

### Identification of existing relevant robotic systems

We identified 40 systems that are considered endoscopic, thoracoscopic, or laparoscopic surgical robots by searching the FDA, EU, Hong Kong, Japan, and Korean databases and investigating the program book of the July 2023 Annual Meeting for the Society of Robotic Surgery. Four devices were identified in the EU database, 14 in the FDA database, 1 in the Hong Kong database, 2 in the Japan database, and 1 in the Korean database. All but 6 of these products were also advertised at the Society for Robotic Surgery Annual Meeting in 2023. Table 1 lists each system with its manufacturing company, primary use as advertised on the company’s official webpage, country of origin, and if applicable, year of FDA, EU, Japanese or Korean market approval when available.

**Table 1:** Currently marketed and/or FDA-approved endoscopic, thoracoscopic, and laparoscopic surgical robots

Robot name	Company	Primary use	Country	Year of regulatory approval
Anovo	Momentis Surgical, Inc	Gynecology	Israel	2023, FDA
Aquabeam	Procept BioRobotics Corp	Urology	USA	2021, FDA; 2022, JPMDA
Avatera	avateramedical GmbH	Gynecology and Urology	Germany	2019, EU
Bitrack	Rob Surgical	General Surgery, Gynecology, and Urology	Spain	N/A
Carina	Ronovo Surgical	Urology	China	N/A
da Vinci	Intuitive Surgical	General Surgery, Otolaryngology, Cardiac, Thoracic, Gynecology, Urology	USA	Model Si: 2005, FDA; 2015 JPMDA Model Xi: 2014, FDA; 2018, JPMDA; 2017, KMFDS Model SP: 2019, FDA; 2017, KMFDS
Dexter	Distalmotion	General Surgery, Gynecology, and Urology	Switzerland	N/A
Edge Medical	Jingfeng Medical	General Surgery, Gynecology, and Urology	China	N/A
Endomaster	Endomaster Pte Ltd	General Surgery	Singapore	N/A
Endoquest	Endoquest Robotics	General Surgery, Otolaryngology, Gynecology, and Urology	USA	N/A
Galaxy	Noah Medical	Thoracic	USA	2023, FDA
Hinotori	Medicaroid, Inc	General Surgery, Gynecology, and Urology	Japan	N/A
Hiwin MTG-H100	Hiwin Technologies Corp	General Surgery, Urology, Gynecology	Taiwan	Not specified, EU
Hugo	Medtronic	General Surgery, Gynecology, and Urology	USA	N/A
ILY	Sterlab	Urology	France	2021, EU
Ion	Intuitive Surgical	Thoracic	USA	2019, FDA
KangDuo	Harbin Sagebot Intelligent Medical Equipment Co., LTD	General Surgery, Thoracic, Gynecology, Urology, Spine Surgery, and Otolaryngology	China	N/A
MARS	Levita Magnetics	General Surgery, Urology	USA	2017, FDA
Mira	Virtual Incision	General Surgery	USA	N/A
Monarch	Ethicon, Johnson & Johnson	Urology, Thoracic	USA	Bronchoscopy model: 2018, FDA Urology model: 2022, FDA
Maestro	Moon Surgical	General Surgery	USA	2022, FDA
Novus Flex	Novusarge	Otolaryngology	Turkey	2018, FDA
Ottava	Johnson & Johnson	General Surgery	USA	N/A
Revo-I	Meere Company	General Surgery, Gynecology, and Urology	South Korea	2017, KMFDS
Senhance	Asensus Surgical US, Inc	General Surgery, Thoracic, and Gynecology	USA	2017, EU + FDA
Sentire	Cornerstone Robotics Ltd	Soft tissue, not otherwise specified	China	N/A
Sirius	Precision Robotics	General Surgery, Thoracic, and Gynecology	Hong Kong	2022, FDA
SoloAssist II	Stryker	General Surgery, Cardiology, Gynecology and Urology	USA	2018, FDA
SSI Mantra	SS Innovations International Inc,	General Surgery, Cardiothoracic, Otolaryngology, Gynecology, and Urology	India	N/A
Surgerii	Shurui Robotics	General Surgery, Cardiothoracic, Pediatrics, Gynecology, and Urology	China	N/A
Symani	Medical Microinstruments, Inc	Plastic Surgery, Orthopedics, Lymphatic Surgery, Nerve Repair Surgery, Otolaryngology, and Pediatric Surgery	Italy	N/A
Toumai	MicroPort Scientific	General Surgery, Urology	China	N/A
Versius	CMR Surgical Ltd	General Surgery, Thoracic, Gynecology, and Urology	United Kingdom	N/A
Vicarious	Vicarious Surgical, Inc	General Surgery	USA	N/A
Virtuoso	Virtuoso Surgical	Thoracic, Neurosurgery, Gynecology, and Urology	USA	N/A
Wego Microhand S	Shandong WEGO Surgery Robot Co., LTD	General Surgery	China	N/A
Zamenix R	Roen Surgical, Inc	Urology	South Korea	2022, KMFDS

*N/A* Not applicable, *FDA* United States Food and Drug Administration 510(k) or premarket approval, *EU* European Union European Commission, *KMFDS* Korean Ministry of Food and Drug Safety, *JPMDA* Japanese Pharmaceuticals and Medical Devices Agency

### Literature search for surgical robotic classification systems

**Fig. 1:**
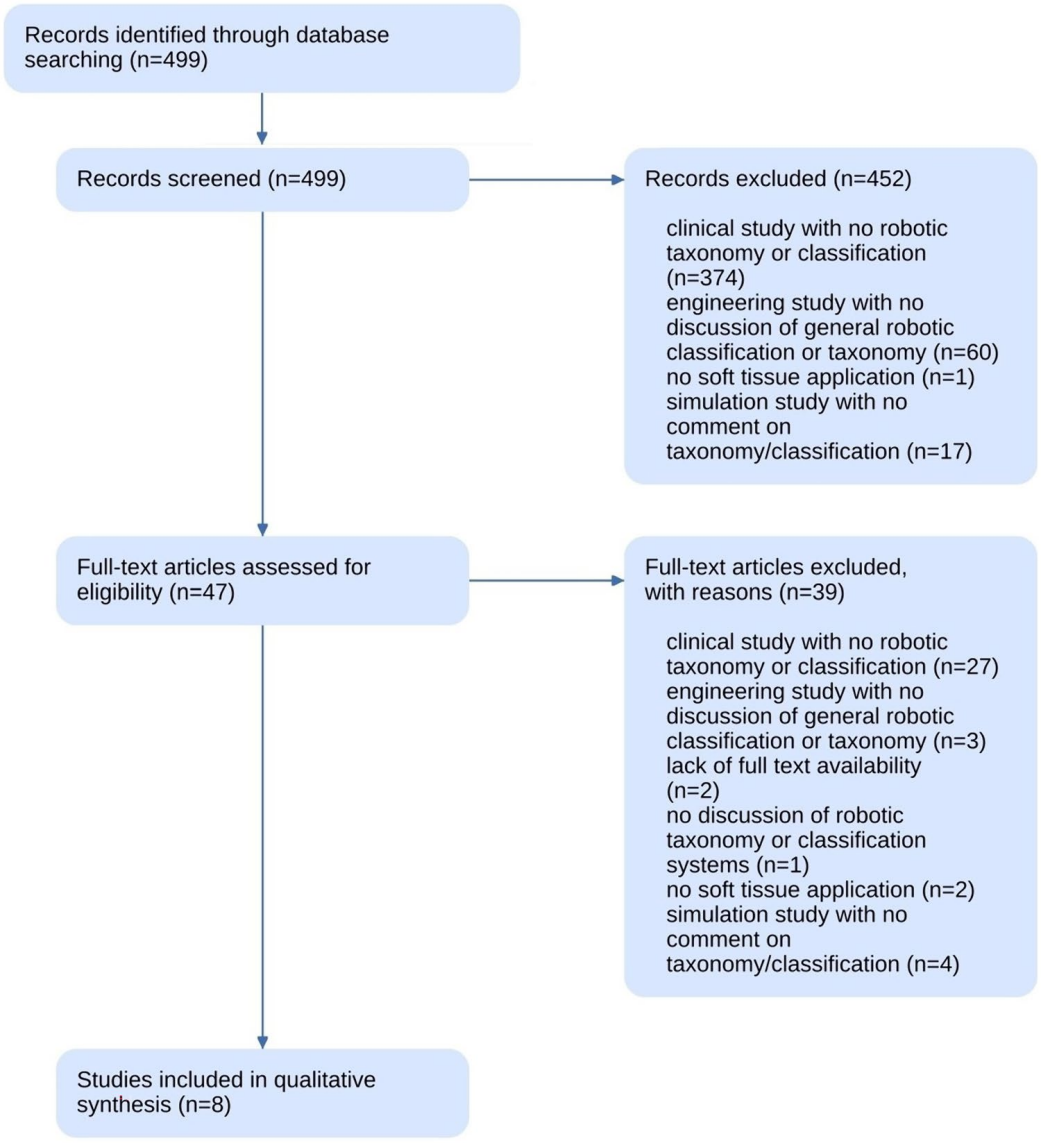
PRISMA flowchart of systematic literature review

Our systematic literature search identified 499 articles that were then screened for relevance. Forty-seven were included for full text review, and 2 records did not have a full text available in the English language. Those which underwent full text review but were excluded as they had no discussion on classifications systems for soft tissue surgical robotics are shown in Fig. 1; reasons included a clinical outcome study (27), education or simulation studies (4), primarily engineering studies (3), no soft tissue application (2), and 1 had no general discussion of classification of systems. Eight studies were ultimately included in the systematic review and are visible in Table 2 [16–23].

**Table 2:** Summary of studies from literature review included in qualitative synthesis

First author, Year, Journal	Publication title	Summary of paper	Contribution to classification created by our committee
Davies, 2000 Proc Inst Mech Eng	A review of robotics in surgery [16]	Classification into: Powered robotics used as possible tool holders, active robots (largely used for biopsy), and master–slave ‘telemanipulator’ systems (such as the Intuitive’s da Vinci)	These classifications were considered largely outdated for today’s surgical robotic field, and all included devices in our classification fall in to the telemanipulator category
Korb, 2004, Int J Oral Maxillofac Surg	Robots in the operating theatre—chances and challenges [17]	Classification into categories by surgical specialty	We chose to remain agnostic to surgical specialty as much crossover exists, and several specialties operate on “soft tissue” rather than bone or endovascular
Camarillo, 2004, Am J of Surg	Robotic technology in surgery: past, present, and future [18]	Division of devices into a role-based taxonomy: 1. Passive role (e.g., CT scanner), 2. Restricted role (e.g., AESOP camera holder), 3. Active role (e.g., da Vinci)	Not considered useful for our committee as the passive role devices are not largely understood as robotic, and our focus was more on architecture than role
Kuo, 2012 Int J Med Robot	Kinematic design considerations for minimally invasive surgical robots: an overview [19]	Designation of surgical robotic arms into 8 categories based on the remote center of motion (i.e., isocenter, belt, spherical linkage)	Considered by our committee to have too much technical focus and less relevant to clinical application
Avgousti, 2016 Biomed Eng Online	Medical telerobotic systems: current status and future trends [20]	Division into short-distance (in same room) and long-distance (increasingly remote) tele-robotics	All current viable products fall into the short-distance model, thus this division was not included
Schleer 2019, Int J comput Assist Radiol Surg	Toward versatile cooperative surgical robotics: a review and future challenges [21]	Classification regarding level of autonomy, resulting in active (some autonomy, such as therapeutic radiation administration), semi-active (a robotic arm that is static and directly controlled), and synergistic (which includes teleoperation)	Most soft tissue robotic devices are synergistic in this classification so this was not included in our scheme
Zhang, 2021 Int J Med Robot	Research progress and development trend of surgical robot and surgical instrument arm [22]	Designation of surgical robot arm into 3 categories based off end-effector arm: rigid, articulated (multiple joints per end-effector), and continuum (continuously flexible like a snake)	This was a basis for our division of rigid versus flexible
Lefkovich 2023, PLoS One	Identification of predicate creep under the 510(k) process: A case study of a robotic surgical device [23]	Specifically describes how the da Vinci has built a case of regulatory clearance by building from foundation of one device into many different FDA product codes of devices	Discusses regulatory classifications within the FDA but does not propose a method to classify robotic devices

### Committee creation of classification

**Fig. 2:**
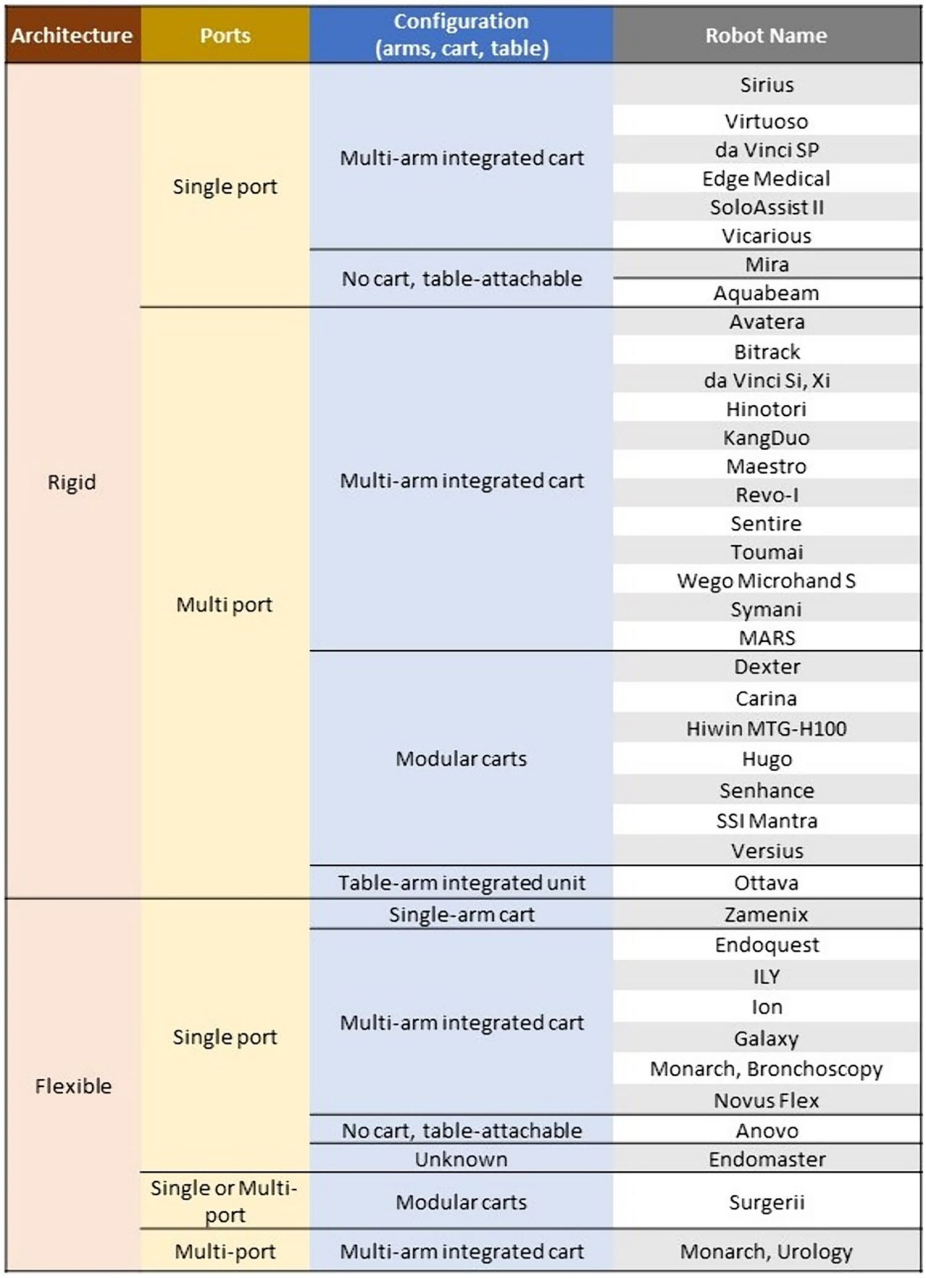
Proposed classification of soft tissue surgical robots

Our proposed classification of robotic systems is depicted in Fig. 2. Our committee participated in an iterative asynchronous process to over several iterations to reach a consensus.

The primary descriptor level in the classification is named *Architecture* and refers to whether the device is rigid or flexible. The committee reviewed the results of the literature search for existing classification systems and felt that the description of a continuum in the Zhang classification was important to include in the proposed classification system [22]. The description of “flexible” refers to robotic arms that have a continuously articulating shaft in a way that is similar to a flexible endoscope, while “rigid” corresponds to robotic arms with articulated joints.

The next level of classification, *Ports*, refers to whether the device is primarily designed for single- or multi-port use, characterizing the number of robotic arms that interact with a trocar or patient entry site during standard operation. The committee acknowledged that some of these devices may have a primary or most common use as a multi-port device but are able to be used in a single-port fashion. For instance, the Intuitive da Vinci Xi has multiple arms that typically interface with multiple trocars, which placed it in the “Multi-Port” Division. On the contrary, the Intuitive da Vinci SP has multiple robotic arms which are designed to enter through a single trocar; this quality placed this device in the “single port” division.

Following this is the classification of *Configuration,* which refers to how the robotic arms are arranged or configured around the device hardware and around the target anatomy. A single arm or multiple working arms may be integrated into a central cart. Modular cart systems exist that can be independently arranged around the operating table each with their own arm. Some systems are not cart-dependent and may be attachable to the table via a clamp. Others are integrated into the operating table.

## Discussion

There has been a steep continuous progress and development in the field of surgical robots, where they are becoming more specialized and integrated to meet the ever-growing list of clinical needs. There are at least 50 surgical robots across all surgical specialties in various stages of development or availability on the market. Given the number and diversity of surgical robots in public discourse, we propose what is, to our knowledge, the first surgical robot classification to better categorize these diverse systems. This will help individuals in the clinical and medical technology fields better understand systems, communicate more clearly about the established potential of robotic surgery, and identify ongoing needs. The goal would be to establish this classification in the field of robotic surgery, and we plan to continue to add and expand to it as we navigate new and emerging breakthrough technologies.

We aimed to develop a universal and generalizable classification to categorize surgical robotic systems based on recognizing and classifying shared traits among these systems. This classification was created in a repeatable, guideline-based fashion that was suited to this task using the NISO framework applicable to fields outside healthcare or biology [7]. These methods are similar to other methods of classification creation that have been published in healthcare literature [6].

We chose to follow this repeatable methodology of creating a classification with the intent of maintaining as much objectivity as possible in the result. However, even a committee is subject to biases and conflicts. For instance, although 2 engineers were on the classification committee, the classification of devices proposed here is largely designed as a description of robotic architecture from a surgeon’s perspective. When considering the features that are included into this classification scheme, the committee focused on the end-effector that interacts with the patient. Similarly, classification of the patient cart was prioritized over the surgeon console. One conflict that appeared in our committee process was whether to include a classification of patient entry approach, whether being via a natural orifice such as endoscopy or via an incision. The committee chose an agnostic approach of not including this distinction, as flexible endoscopes can be inserted through incisional trocars, and similarly, rigid robotic devices can operate within the oral or anal cavities.

While we attempted to keep these categorizations as binary as possible, there are certain systems that defy some of the strict definitions, and it would be nearly impossible to account for these discrete variations in detail in what we aimed to be a widely generalizable classification. For example, when discussing rigid and flexible sheaths it is notable that certain systems have rigid sheaths but flexible and articulating end effectors (example: Vicarious), while others have purely rigid sheaths up to the level of the instrument’s wrist (example: MMI’s Symani). These classifications have been previously established, and if some devices have both flexible and rigid characteristics, we would categorize the device with a majority vote [22]. Regarding the number of patient ports, this categorization describes the most common use. That said, some systems may have a single trocar and multiple working arms (example: Intuitive da Vinci SP), and others may have multiple trocars and working arms (example: Medtronic Hugo). Additionally, some systems may have multiple trocars entering the body cavity through the same incision, by the use of a gel port for example, whereas others require individual incisions for each trocar.

In regard to creating a complete list of soft tissue surgical robots, it is important to note that the information presented here is entirely based on the information that is publicly available, either through the FDA database, national and international conferences, company websites, and PubMed. It does not include device applications that are pending approval, as this is not public information. It is likely that there are several other systems not captured here as they may have not yet publicized their work or may be in the germinal phase of research and development, among other reasons. We also note that our search for surgical robotic devices did not include the Chinese National Medical Products Administration as this body does not have a publicly searchable database of approved products. It also does not include Korean MFDS-approved devices predating 2014, as this information is not publicly available on their website.

Despite the limitations aforementioned, our review of surgical systems proposes a classification scale for identifying current and emerging surgical robotic technologies. Notably, this categorization is very contemporary and will be outdated as new technologies emerge. As surgical robotics evolves over time, this nomenclature will likely need to adapt and grow along with the technology it describes.

The standardized common language and classification presented here could be useful to several groups of users. Surgeons and proceduralists need a framework to understand the large variety of robotic-assisted surgical devices and what procedures each may be of best use. Healthcare administrators need this information to plan budgets, grow and manage practices, and monitor patient quality outcomes. Such classification would be of particular use when outfitting operating rooms and deciding which robotic devices best suit their practice in accounting for caseloads. Engineers can use this classification to better understand the device design from the clinical perspective of a healthcare provider. This would also allow engineers to better identify and address unmet needs in the industry. Surgical educators need to understand the organization of these devices to keep up with the development of the technology as they train future surgeons. Regulatory agencies could benefit from adopting a common language to keep up with future innovations. Surgical societies, by creating guidelines, holding meetings, and publishing research, can standardize language on surgery-related topics. Adopting this common language would facilitate communication about robotic surgery and allow community members to keep current on the practice of evidence dissemination regarding surgical robotics and innovation. This multidisciplinary communication and collaboration will only help further education and development in robotic surgery.

## Conclusion

We propose what is, to our knowledge, the first multi-level classification for surgical robotic systems. We aim to create a unified language to assess, evaluate, and communicate about existing and evolving robotic technologies. This classification will facilitate the understanding of future innovations of surgical robotics across the disciplines of healthcare, industry, academics, and regulatory bodies.
